# Establishment of an in Vitro Human Blood-Brain Barrier Model Derived from Induced Pluripotent Stem Cells and Comparison to a Porcine Cell-Based System

**DOI:** 10.3390/cells9040994

**Published:** 2020-04-16

**Authors:** Annalise Di Marco, Domenico Vignone, Odalys Gonzalez Paz, Ivan Fini, Maria Rosaria Battista, Antonella Cellucci, Elena Bracacel, Giulio Auciello, Maria Veneziano, Vinod Khetarpal, Mark Rose, Alessandro Rosa, Isabelle Gloaguen, Edith Monteagudo, Todd Herbst, Celia Dominguez, Ignacio Muñoz-Sanjuán

**Affiliations:** 1 High content Biology and Screening, IRBM SpA, 00071 Pomezia, Italy; D.Vignone@irbm.com (D.V.); o.gonzalez@irbm.com (O.G.P.); i.fini@irbm.com (I.F.); M.Battista@irbm.com (M.R.B.); a.cellucci@irbm.com (A.C.); g.auciello@irbm.com (G.A.); i.gloaguen@irbm.com (I.G.); 2 Drug Metabolism and Pharmacokinetics, IRBM SpA, 00071 Pomezia, Italy; e.bracacel@irbm.com (E.B.); m.veneziano@irbm.com (M.V.); e.monteagudo@irbm.com (E.M.); 3 CHDI Management/CHDI Foundation, 6080 Center Drive, Los Angeles, CA 90045, USA; Vinod.Khetarpal@chdifoundation.org (V.K.); Mark.Rose@chdifoundation.org (M.R.); Todd.Herbst@chdifoundation.org (T.H.); Celia.Dominguez@CHDIFoundation.org (C.D.); 4 Department of Biology and Biotechnology Charles Darwin, Sapienza University, Piazzale Aldo Moro 5, 00185 Rome, Italy; alessandro.rosa@uniroma1.it; 5 Center for Life Nano Sciences, Istituto Italiano di Tecnologica, Viale Regina Elena, 291, 00161 Rome, Italy

**Keywords:** blood brain barrier, human induced pluripotent stem cells, CNS, permeability, Huntington’s disease

## Abstract

The blood-brain barrier (BBB) is responsible for the homeostasis between the cerebral vasculature and the brain and it has a key role in regulating the influx and efflux of substances, in healthy and diseased states. Stem cell technology offers the opportunity to use human brain-specific cells to establish in vitro BBB models. Here, we describe the establishment of a human BBB model in a two-dimensional monolayer culture, derived from human induced pluripotent stem cells (hiPSCs). This model was characterized by a transendothelial electrical resistance (TEER) higher than 2000 Ω∙cm^2^ and associated with negligible paracellular transport. The hiPSC-derived BBB model maintained the functionality of major endothelial transporter proteins and receptors. Some proprietary molecules from our central nervous system (CNS) programs were evaluated revealing comparable permeability in the human model and in the model from primary porcine brain endothelial cells (PBECs).

## 1. Introduction

BBB models are valuable tools for studying mechanistic aspects of drug transport to the brain, as well as for developing strategic solutions to circumvent the BBB and allow passage of CNS therapeutics. These platforms should be physiologically relevant and able to assess the responses to experimental treatments or stimuli otherwise difficult to reproduce or to dissect out in vivo [1]. Current methodologies do not allow the full reproduction of the physiology of the BBB in vivo in a single platform, but recent advances have enabled the development of in vitro BBB models able to reproduce most of the functionality of the BBB.

We have previously demonstrated the usefulness of an in vitro porcine BBB model in drug screening for Huntington’s disease (HD) with a good correlation with in vivo data obtained in rodents [2]. The porcine model displayed various BBB features such as high TEER and low paracellular permeability, expression of tight junction proteins, polarized expression, and functionality of the major efflux transporters. Transcriptomic, proteomic, and functional studies indicate that there are differences between the human and other mammalian BBB that further complicate predictions on drug uptake in humans, based on preclinical animal species [3,4]. However, differences in the BBB among species suggested that human BBB models could complement the results obtained in animals and have the potential to better mimic the in vivo conditions for a higher translatability. Given the high attrition rate of CNS drugs in clinical trials, an in vitro human BBB model is likely to be more suitable for optimizing CNS drug selection and to predict human brain exposure.

Recent scientific advances in stem cell research have opened new opportunities for in vitro disease modelling and stem cells have been explored to develop functional in vitro human BBB systems [5,6]. Additionally, reprogrammed stem cells obtained from patients provide the opportunity to study the BBB functionality in the disease context, taking into account the evidence that the neurodegenerative processes could be highly associated with a significant dysfunction of the BBB [7,8].

Although new approaches such as microfluidic systems capable of forming a three-dimensional configuration [9] have emerged as promising human BBB models, they are generally not yet compatible with screening activities. The simplest and most feasible in vitro BBB transport model consists of a monolayer of cells seeded on a permeable support. Analogous to our previously published porcine model, we used the Transwell apparatus to grow human endothelial cells derived from iPSCs on microporous semipermeable inserts. The iPSCs could acquire a brain endothelial phenotype under a spontaneous differentiation in unconditioned medium protocol. The iPSC-derived endothelial cells showed reduction of the pluripotency markers during the differentiation process along with increased expression of endothelial markers and tight junction proteins. They were characterized for their ability to form a tight barrier with a high TEER value and low permeability for paracellular markers. The permeability values of chemically different compounds indicated that the major BBB transporters like PGP or BCRP were expressed and functional. The possibility to cryopreserve the endothelial precursors as a source of terminally-differentiated cells, with no loss of BBB characteristics, enhanced both the reproducibility and the scalability of the hiPSC-derived BBB model. Commercial and investigational compounds against different CNS targets revealed comparable permeability in the human and porcine models.

## 2. Materials and Methods

### 2.1. Reagents

Fetal bovine serum, penicillin/streptomycin, glutamine, HEPES, fungizone, deoxyribonuclease I, and Hoechst 33342 were from Invitrogen (Thermo Fisher, Monza Italy). [14C]sucrose, [14C]mannitol, [3H]methyl-glucose, [14C]leucine, [14C]phenytoin, [14C]caffeine, [3H]digoxin, [3H]daunomycin, [3H]testosterone, [3H] [D-Ala2]-deltorphin II, [3H]propranolol, [3H]prazosin and Microscint 20 scintillation liquid were purchased from Perkin Elmer, Milan Italy and [3H]vinblastine, [14C]Taxol, [3H]methotrexate, [3H]gabapentin and [3H]flumazenil were from BIOTREND Chemikalien GmbH (Köln, Germany).

Fluorescein isothiocyanate-labelled FITC-dextran 40, dimethyl sulfoxide (DMSO), lucifer yellow, rhodamine-123, kynurenic acid, elacridar (GF120918), Y-27632 (ROCK inhibitor), and triton X100 were purchased from Sigma–Aldrich, Milan, Italy, and 8% paraformaldehyde aqueous solution was from Electron Microscopy Sciences (Hatfield, PA, USA).

CHDI compounds, denoted cp (A to J) and histone deacetylase (HDAC) inhibitor (cp K) were synthesized as previously described [10,11].

### 2.2. Karyotype

The karyotyping using the Q-banding method at passages 19 and 27 (Cl1) and passages 30 (Cl2), with a 350 band resolution was performed by the ISENET Biobanking service unit in Milan, Italy (www.isenetbiobanking.com).

### 2.3. Flow Cytometry

Singularized cells were fixed and permeabilized with a commercial fixation buffer: Transcription Factor Buffer Set (BD Pharmingen™) according to the manufacturer’s protocol. Cells were incubated with human Oct-4A, Von Willebrand Factor antibody or isotype control antibodies (see Table S1). The cell suspension was analyzed on a BD FACS Canto II Flow cytometer. Isotype-match cells were used as control. Data were analyzed with FCS Express software (version 5.0.85).

### 2.4. Embryoid Bodies (EB) Formation

iPSCs were singularized with Accutase, seeded in 100 µL of mTeSR containing 10 µM Rho-associated kinase (ROCK) inhibitor Y-27632 in Corning spheroid microplate 96-well ultralow attachment (1500 cells /well) and allowed to settle by centrifugation at 100× *g* for 2 min. Medium was changed the day after. The EB have been cultured for seven days with daily medium change.

### 2.5. Derivation of Brain Microendothelial Cells from Human iPS Cells

The iPSC line used in this study is described by Lenzi et al. [12] and thereafter named clone 1 (Cl1). The second one named clone 2 (Cl2) (Cell line ID - NN0004300) comes from RUCDR Infinite Biologics (iPS Academia Japan, Inc., Kyoto, Japan). Cells were maintained on Matrigel (Corning from Sigma-Aldrich, Milan, Italy)-coated surfaces in mTeSR1 (STEMCELL Tech, Cambridge, UK), as previously described [13] and passaged with 1 mg/mL dispase (Thermo Fisher, Monza, Italy) roughly every four days for a maximum of 33 passages. The protocol used was a modification of the protocol described by Lim et al. [7], adapted from Lippmann et al. [5].

Briefly, iPSC colonies were dissociated as small aggregates with with ReleSR (STEMCELL Tech, Cambridge, UK) and plated onto Matrigel-coated plates in mTeSR1. After 2–3 days, culture medium was switched to Unconditioned Medium (UM): 80% Knockout DMEM/F12 and 20% KnockOut Serum Replacement (KOSR), containing GlutaMAX 1.6X, NEAA 1X, β-mercaptoethanol 0.11 mM, and penicillin-streptomycin 0.1X (all from Thermo Fisher, Monza, Italy) with medium change every day. After six days culture medium was replaced with human endothelial cell medium (hEC) (human endothelial serum-free cell medium, Thermo Fisher, Monza, Italy) containing 20 ng/mL bFGF (STEMCELL Tech, Cambridge, UK) and 1% platelet-poor plasma-derived bovine serum (PDS) (Thermo Fisher, Monza, Italy) for BMEC colony expansion and maturation for two days. During this time, the samples were treated with 10 µM retinoic acid (RA, Sigma-Aldrich, Milan, Italy). Cells were then plated in medium without RA onto human placenta derived collagen-IV (Sigma-Aldrich, Milan, Italy) and human plasma derived fibronectin (Thermo Fisher, Monza, Italy) coated tissue culture plates or 12 well Transwell filters (1.12 cm^2^ growth area, 0.4 µm pore size, Corning from Sigma-Aldrich, Milan, Italy) for 24 h. TEER was measured to confirm efficient endothelial differentiation. The cells were kept in culture in hEC medium with 1% PDS without bFGF and co-culture was started with human astrocytes. When TEER increased, permeability studies were performed. Human-induced pluripotent stem cell derived brain microvascular endothelial cells will be referred as hiPSC-derived BMECs.

### 2.6. Cryopreservation and Thawing of hiPSC-Derived BMECs

Cells were cryopreserved as previously reported [14]. Briefly, at D8 of differentiation, the cells were dissociated with Accutase (Sigma–Aldrich, Milan, Italy) to obtain a single-cell suspension and resuspended in hEC medium containing 10% DMSO (Sigma–Aldrich, Milan, Italy), 30% PDS, and 10 µM Y-27632. The cryotubes were placed overnight at ‒80 °C in an isopropanol container before definite storage in liquid nitrogen. 

Upon thawing at 37 °C, the cells were resuspended in hEC medium with 1% PDS, containing 20 ng/mL bFGF and 10 µM Y-27632, and seeded at a density of 1 million cells/cm^2^ on 12 well Transwell inserts or 0.5–1 million cells/cm^2^ in tissue culture plates (previously coated with collagen IV/fibronectin as described in paragraph 2.5). After 24 h, culture medium was changed and protocol continued as for fresh cells.

### 2.7. Culture of Astrocytes

Cryopreserved human astrocytes from ScienCell Research Laboratories (Carlsbad, CA, USA) were directly seeded at the bottom of 12-well plate coated with 2 µg/cm^2^ poly-l-lysine (ScienCell Research, Carlsbad, CA, USA) at 5 × 10^3^ cells/cm^2^. The astrocyte medium (ScienCell Research, Carlsbad, CA, USA) was renewed after 24 h to eliminate DMSO. After 24 h, cells were put in co-culture with hiPSC-derived BMECs. Prior to co-culture, astrocytes were characterized for the expression of Glial fibrillary acidic protein (GFAP) by immunofluorescence. Astrocytes from different lots were used with an expression of GFAP > 80%.

### 2.8. PBECs Based Model

Isolation of PBECs and the set up for the transport assay were performed as previously described [2].

### 2.9. Immunocytochemistry

Cells in Transwell inserts (polyester membrane Transwell-Clear) were washed with phosphate-buffered saline (PBS) and fixed in 4% paraformaldehyde for 20 min at room temperature (with the exception of PGP for which cold methanol (VWR Chemicals, Milan, Italy) fixation was used). Cells were permeabilized by washing with PBS/0.1% Triton X-100 and blocked in PBS containing 1% bovine serum albumin (blocking buffer) for 2 h at room temperature. Primary antibodies, diluted as reported in Table S1, were added in blocking buffer for 2 h at room temperature. Cells were then incubated in blocking buffer with the secondary antibody, diluted as recommended by the manufacturer, and the nuclear stain Hoechst 33,342 (2 µM) for one hour at room temperature. Cells were also stained using the three-germ layer immunocytochemistry kit (Thermo Fisher, Monza, Italy) following the manufacturer’s instructions. All cell images were acquired with an INCell Analyzer 2000 (GE Healthcare, Rome, Italy) and analyzed with IN Cell Developer Toolbox software version 1.9.1.

### 2.10. Western Blot

hiPSC-derived BMECs were harvested from Transwell inserts (d1) by trypsinization (Trypsin-EDTA, Life Technologies, Monza, Italy), primary human brain microvascular endothelial cells (HBMVECs, iXCell Biotechnologies, California USA) were thawed in MEM 1X. Cells were lysed in RIPA buffer (300 mM NaCl, 10 mM Tris HCl_pH 8.0, 10 mM KCl, and 1 mM EDTA) containing 1% nonidet p-40, 1% sodiumdeoxycholate, 0.1% sodium dodecyl sulphate (SDS), 1 mM phenylmethylsulfonyl fluoride (PMSF), and protease inhibitor cocktail (Roche). Lysates were sonicated on ice with a Branson 450 sonicator, cleared by centrifugation and extracts (20 µg) were separated by SDS-PAGE on 4%–12% bis-tris gel (Life Technologies, Monza, Italy). After transfer onto a nitrocellulose membrane (Whatman, Sigma–Aldrich, Milan, Italy) and blocking in 5% milk in TBST (150 mM NaCl, 10 mM Tris HCl_pH 7.5, and 0.05% Tween 20), primary antibodies (see Table S1) were incubated over night at 4 °C. After incubation with the dye-conjugated secondary antibodies protein detection was achieved using Infrared Odyssey system (LiCor) software version 2.1.

### 2.11. mRNA Extraction and Quantification by Real-Time qRT-PCR (TaqMan)

Total RNA was extracted after cell lysis using an RNA extraction kit (Macherey-Nagel, Düren, Germany) on a Microlab STARlet (Hamilton, NV, USA), according to the manufacturer’s protocol. Quantitative analysis of specific mRNA expression was performed by real-time qRT-PCR, by subjecting the resulting cDNA to PCR amplification using 384-well optical reaction plates in the QuantStudio 12k Flex Real-Time PCR (Applied Biosystem, Foster City, CA, USA). The thermocycling conditions were initiated at 48 °C for 15 min with an enzyme activation step of 95 °C for 10 min, followed by 40 PCR cycles of denaturation at 95 °C for 15 s and anneal/extension at 60 °C for 1 min. Single gene real-time PCR primers and probes were purchased from Applied Biosystem, California USA as “TaqMan Gene Expression assay”. Genes evaluated by TaqMan are reported in Table S2. The housekeeping gene glyceraldehyde-3-phosphate dehydrogenase (GAPDH) was used for normalization. Relative differences in mRNA expression were determined by QuantStudio 12k Flex software (version v.1.2.3) based on threshold cycle (Ct) data of the target gene versus an endogenous control gene for each reaction.

To evaluate the self-renewal capacity and the pluripotency as well as the trilineage differentiation potential, mRNA was subjected to the hPSC scorecard assay (Thermo Fisher, Monza, Italy) after RNA extraction with RNeasy mini kit (Qiagen, Hilden, Germany) for iPSC colonies. cDNA was obtained with a high capacity cDNA reverse transcription kit (Thermo Fisher, Monza, Italy) and qRT-PCR was performed in QuantStudio 12K Flex as above. Results were analyzed with the hPSC Score Card Panel software.

### 2.12. Transcriptome Analysis

hiPSC-derived BMECs were lysed at d1 co-culture in supplied RLT buffer containing 1% β-mercaptoethanol. Total RNA was extracted with the RNeasy mini kit (Qiagen, Hilden, Germany) following the manufacturer’s protocol. Samples were purified from genomic DNA by an additional step of DNAse I digestion (DNase Max KIT from Qiagen, Hilden, Germany). RNAseq was performed by the provider (Genomnia srl, Bresso, Italy), using Ion AmpliSeq technology, allowing the evaluation of over 20,000 genes. The differential analysis was performed with the R edgeR package (version 3.20.9) [15]. The output value for the detected expression is the normalized counts per million (CPM) value. Data were deposited at ENA’s Sequence read archive with access number PRJEB35916.

### 2.13. TEER Measurement and Permeability Assay

Measurement of TEER, permeability studies and derivation of permeability coefficients were carried out as reported [2]. In each filter a paracellular marker (radiolabeled sucrose, FITC-dextran 40 or lucifer yelow) was added as internal control of the tightness of the monolayer. The amounts of radiotracer and fluorescent tracers were determined by liquid scintillation (Top Count-NXT, Microplate Scintillation and Luminescence counter from Perkin Elmer, Milan Italy) and fluorescence spectrophotometry (Microplate Fluorescence reader, SAFIRE TECAN, Männedorf, Switzerland), respectively, whereas for all other compounds the concentration was determined by LC-MS/MS as described in Di Marco et al [2]. In efflux and influx transport assays, before the addition of the compounds, filters were pre-incubated for 15 min at 37 °C with or without inhibitors and/or substrates: 2 µM elacridar (PGP inhibition), 10 µM JPH203 (LAT-1 inhibition), and 200 µM of phloretin (GLUT inhibition).

### 2.14. Transcytosis Assay

The anti-transferrin receptor antibody MEM-189 (ab1086, RRID:AB_297535) from Abcam, (Milan, Italy) was incubated (3 µg/mL) in HBSS-20 mM Hepes pH 7.4 containing 2% PDS for 2 h and 4 h, at 37 °C and 5% CO_2,_ and on ice at 4 °C. At the end of the incubation, aliquots from both compartments were collected, and after two washes the cells were lysed in 250 µL of cold RIPA buffer. After centrifugation, the supernatants were stored at −20 °C before quantification. The concentration of sodium azide, used as preservative for the antibody stock solution, was equal to 0.0001% in the transport experiment.

Antibody concentrations were measured by a sandwich ELISA. Briefly, AffiniPure goat anti-mouse IgG Fc fragment specific antibody (catalog no. 115-005-164, RRID:AB_2338458, Jackson ImmunoResearch Labs, West Grove, PA, USA) was coated overnight at 0.1 µg/well at 4 °C. After blocking in TBS, containing 0.5% of Tween 20 and 5% milk for 2 h at RT, different dilutions of samples were added to the wells, as well as known concentrations of the measured antibody to create the standard curve. A biotin-conjugated detection antibody (catalog no. 315-066-047, RRID:AB_2340102, Jackson ImmunoResearch Labs, West Grove, PA, USA) was, in turn, measured by a peroxidase conjugated streptavidin (catalog no. 016-030-084, RRID:AB_2337238, Jackson ImmunoResearch Labs, West Grove, PA, USA). The product of the reaction with the TMB substrate, stopped by sulfuric acid, was detected by spectrometry, subtracting the absorbance at 620 nm from the absorbance at 450 nm. Concentrations were determined using the standard curve fitted with a four-parameter regression curve using XLfit 5.5.0.5.

### 2.15. Statistical Analysis

Statistical analyses were performed using an unpaired, two-tailed Student’s t-test for comparisons between two groups. The value of *p* < 0.05 was taken as the criterion for statistically significant difference.

## 3. Results

### 3.1. Stem Cell Characterization

**Figure 1:**
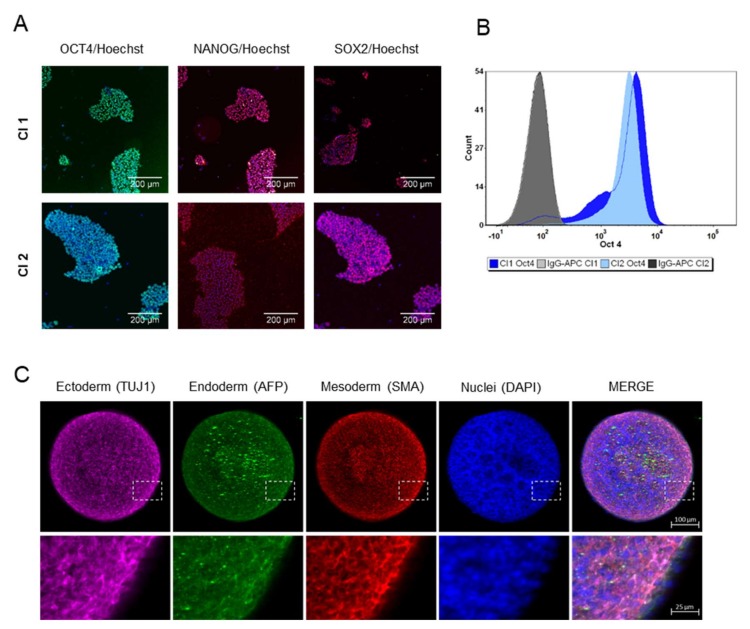
Characterization of human iPSCs. (**A**) Immunofluorescence staining demonstrates the expression of the pluripotent transcription factors OCT4, NANOG, and SOX2 in growing iPSCs (Cl1 and Cl2). Scale bar represented 200 µm. (**B**) Representative flow cytometry analysis of OCT4 positive cells in iPSCs (Cl1-92% and Cl2-98%) during routine culture. (**C**) Expression of germ layer markers in seven-day EBs (Cl1): representative immunofluorescence staining showing the expression of ectoderm germ-layer in pink (TUJ1), endoderm germ layer in green (AFP), mesoderm germ-layer in red (SMA) and nuclei in blue (DAPI), in the single images as well as in the merge view. Magnifications (4×) of the delineated insert are reported. Scale bar represented 100 µm.

The iPSC line used in this study and named clone 1 (Cl1) is described by Lenzi et al. [12], while the cell line referred as clone 2 (Cl2) comes from RUCDR Infinite Biologics (cell line ID: NN0004300). Karyotyping was performed every 10–15 passages by Q-banding analysis. Cells showed no karyotype abnormalities or chromosomal aberration with passages (Figure S1A). Pluripotency was assessed using the TaqMan hPSC Scorecard panel based on RT-qPCR and by immunostaining. As highlighted in Figure S1B, iPSCs expressed self-renewal genes and were not committed to any germ layer. Figure 1A showed how the cells formed typical dense colonies and expressed the transcription factors OCT4, NANOG, and SOX2 characteristic of the pluripotent state [16]. In particular, the expression of OCT4 (Figure 1B) was analyzed by flow cytometry at the beginning of routine culture and used as a quality control for each cell bank that was generated. The iPSC colonies, characterized by high nucleus-to-cytoplasm ratio, formed compact colonies, distinguishable by their characteristic embryonic stem-cell morphology with (Figure 1A). To explore the potential of the iPS cells to undergo terminal differentiation in vitro to cells of the ectoderm, mesoderm, and endoderm lineages, cells were grown in suspension to induce their spontaneous aggregation in embryoid bodies (EBs). After seven days the germ layer markers of ectoderm (TUJ1), mesoderm (SMA), and endoderm (AFP) were expressed, although a clear pattern formation or highly organized structures were not observed (Figure 1C), typical of older EBs [17].

### 3.2. iPSCs Differentiation into Endothelial Cells

**Figure 2:**
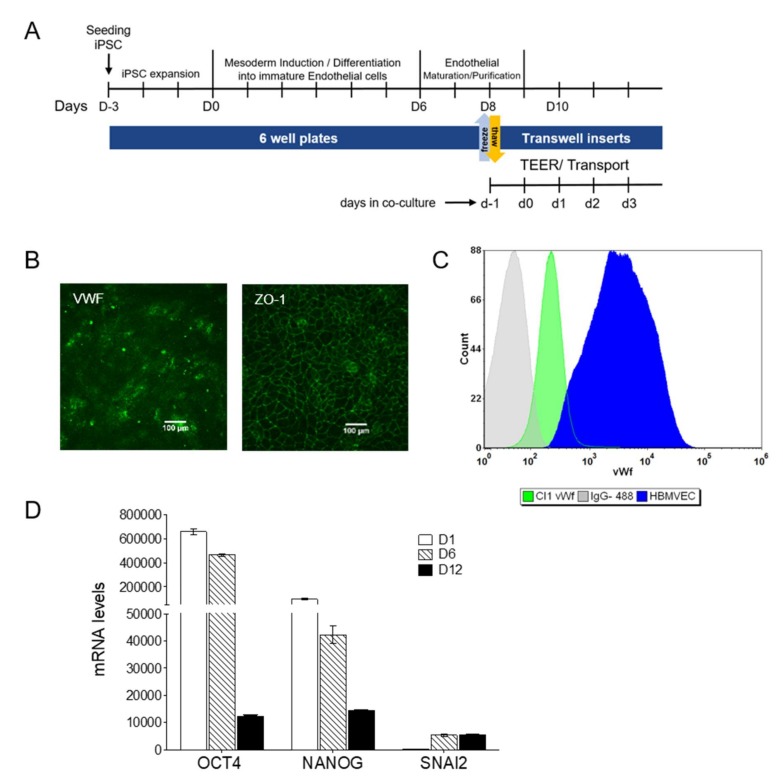
Generation and selection of hiPSC-derived BMECs. (**A**) Scheme illustrating the timeline and the cell culture steps. (**B**) Immunostaining of VWF and ZO-1 at D8. Scale bar represented 100 µm. (**C**) Representative flow cytometry analysis of VWF in endothelial progenitors at D8 and HBMVEC cells, used as positive control. The corresponding isotype control was also included. (**D**) Mesodermal transition: mRNA levels for pluripotency and the mesoderm specific marker SNAI2 measured during differentiation. Results (as mean ± sd) are from a representative experiment performed on biological duplicates, each with two technical replicates.

The iPSCs were able to differentiate into mature endothelial cells in about 12 days, as reported in Figure 2A, following the method described in experimental procedures. After eight days of differentiation (D8), before final seeding on Transwells, the expression of the endothelial marker von Willebrand Factor (VWF) and the tight junction protein ZO-1 (Figure 2B,C), confirmed the successful transition of iPSCs into cells with an endothelial phenotype.

During differentiation, the mRNA expression of the pluripotency markers OCT4 and NANOG decreased consistently, while the expression of the mesodermal lineage marker SNAI2 reached a plateau at D6, before the pluripotency markers were lost (Figure 2D).

**Figure 3:**
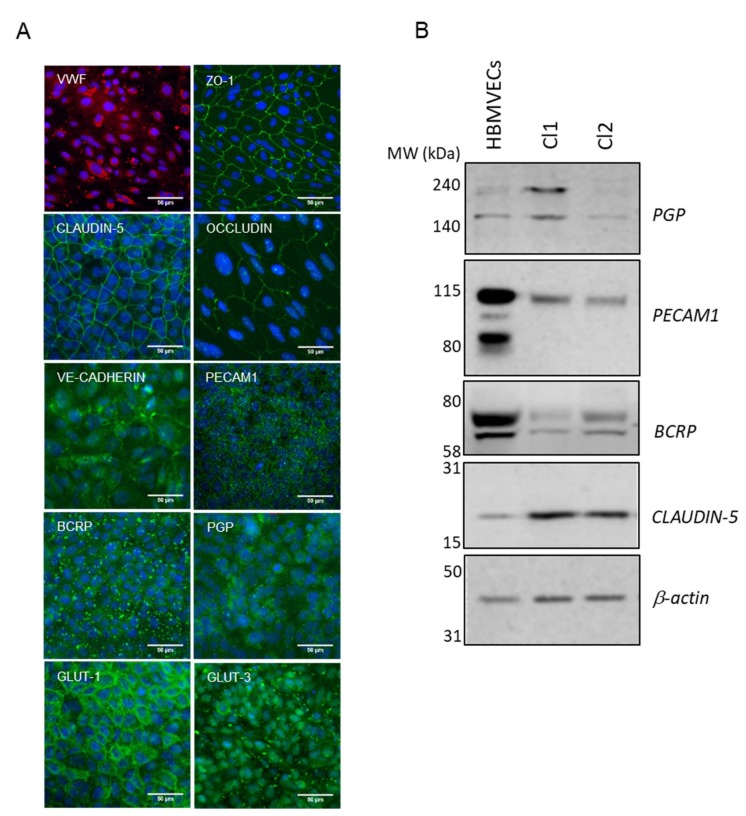
Final differentiation of hiPSCs into brain endothelial cells. (**A**) Representative immunofluorescence staining, at d1 co-culture in Transwell filters (Cl1), demonstrating the expression of endothelial relevant proteins: VWF, ZO-1, CLAUDIN-5, OCCLUDIN, VE-CADHERIN, PECAM1, BCRP, PGP, GLUT-1, and GLUT-3. Nuclei were counterstained with Hoechst (blue). Scale bar represented 50 µm. (**B**) Representative cropped western blot confirming the expression of CLAUDIN-5, PECAM1, BCRP, and PGP in hiPSC-derived BMECs compared to primary HBMVECs. β-actin was used as the loading control.

The final differentiation into mature hiPSC-derived BMECs was completed by culture on collagen/fibronectin-coated culture plates or Transwell inserts. The results from Lippmann’s group demonstrated the importance of co-culture with astrocytes in TEER increase [6]. Although some authors reported differentiation in the absence of supporting cells [14,18,19,20], the group of Zhang [21] demonstrated how co-culture with astroglia ameliorates the barrier phenotype, increasing TEER and reducing LY permeability. For our transport studies, and similarly to Mabondzo’s group [22] who clinically validated the system, we used only astrocytes as it has been reported that neither TEER nor permeability characteristics gained benefit from the presence of pericytes [23]. At D10 of the differentiation process, in the presence of human astrocytes, (d1 co-culture in Transwell filters, following the nomenclature as reported in Figure 2A), hiPSC-derived BMECs were characterized by immunofluorescence staining for selected BBB markers (Figure 3A (Cl1) and Figure S2A (Cl2)). The endothelial marker VWF was expressed with the characteristic cytoplasmic Weibel–Palade bodies’ localization. Cells formed well-developed tight junctions expressing ZO-1, OCCLUDIN, CLAUDIN-5, and the cell-cell adhesion protein CD31 (PECAM1). The adherence junction protein VE-CADHERIN, reported to play an important role in endothelial junction integrity, was also abundantly expressed [24].

The hiPSC-derived BMECs expressed the efflux transporters PGP and BCRP (Figure 3A (Cl1)), which play a critical role at the BBB to prevent the entry of harmful molecules into the brain [25]. Members of the solute carrier family transporters (SLCs) such as GLUT-1 (SLC2A1), the major glucose transporter at the BBB and GLUT-3 (SLC2A3) were also expressed [26,27] (Figure 3A (Cl1) and Figure S2 (Cl2)).

**Figure 4:**
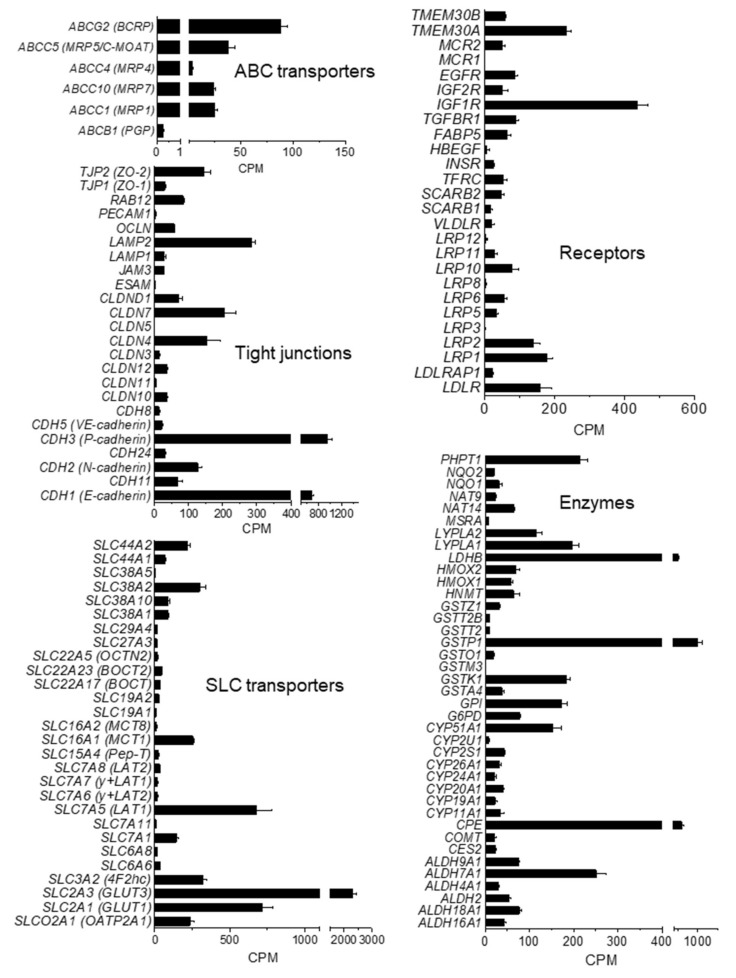
Transcription level of selected genes in iPSC-derived BMEC. mRNA levels at d1 co-culture, as counts per million (CPM), are expressed as mean ± SEM from two different rounds of differentiation of Cl1 and RNA analysis, each with duplicate technical determinations of biological triplicates.

To better characterize the molecular nature of hiPSC-derived BMECs in co-culture with human astrocytes (at d1 co-culture), we performed whole genome expression analysis by RNA sequencing. To facilitate the analysis, selected genes were grouped by their presumed function and orthology (Figure 4, ENA’s Sequence read archive access number PRJEB35916). Of the 52 families of SLC transporters, more than 60% were detected in the hiPSC-derived BMECs model at low, moderate, and high levels. Among the solute carriers, SLC2A3 (GLUT-3) showed the highest expression, followed by SLC7A5 (LAT1) and SLC2A1 (GLUT-1). Kurosawa et al. [20] also reported a higher mRNA expression of GLUT-3 than GLUT-1 (129 vs. 17) in IMR90-derived BMECs by qRT-PCR. Our data were also in agreement with results published by Park et al. [28], where in a human BBB reconstituted in an organ chip microfluidic device, GLUT-3 protein was about three to four-fold more expressed than GLUT-1.

Transcripts for amino-acid and peptide transporters were also detected: SLC1A3 (glutamate, aspartame transporter), SLC3A2, SLC6, SLC15A4, and SLC19A2, although the highest expression was observed for SLC7A5 (LAT-1). Among the SLC16 family of monocarboxylate transporters (MCT), the monocarboxylate transporter-1 (SLC16A1; MCT1) and -8 (SLC16A2; MCT8) were expressed. MCT1 catalyzes the proton-linked transport of monocarboxylates, such as L-lactate across the plasma membrane, whereas MCT8 is essential for thyroid hormone transport across the BBB and it has been reported to be implicated in Allan–Herdon–Dudley syndrome (AHDS), an X-linked disorder characterized by neuropsychomotor abnormalities [29]. Other important transporters for different chemical families such as organic anions (SLCO2A1) or cations (SLC22A) were also expressed.

The hiPSC-derived BMECs expressed the BBB drug efflux transporters P-glycoprotein (PGP/ABCB1), breast cancer-resistant protein (BCRP/ABCG2), and several members of ABCC subfamilies such as the multidrug resistance-associated proteins 1 (MRP1/ABCC1), 4 (MRP4/ABCC4), and 7 (MRP7/ABCC10). Among efflux transporters, BCRP was the most abundant, as previously reported for PBECs [30]. The iPSC-derived BMECs also expressed important transcytosis receptors at the BBB such as the insulin receptor (INSR), low density lipoprotein receptor-related protein 1 (LRP1) and transferrin receptor 1 (TFRC) consistent with published data [31].

The analysis of the intercellular junction complex provided insights into the specialized restrictive microvascular barrier. Tight junction transcripts included members of zonula occludens (ZO) and the claudin family (CLDN). Although RNA levels of CLAUDIN-5 remained almost undetected, protein expression was observed by immunofluorescence (Figure 3A (Cl1) and Figure S2A (Cl2)) and western blot (Figure 3B), which indicated that the protein might have been sufficiently expressed after the acquisition of the BBB phenotype. This is in-line with the data published by other authors [8], where CLAUDIN-5 measured by RNAseq was much less expressed than other tight junctions proteins such as OCCLUDIN or ZO-1 in the hiPSC-derived BMECs lines analyzed, while monolayers displayed proper junctional localization of CLAUDIN-5 by immunocytochemistry.

### 3.3. Drug Transport Studies

**Figure 5:**
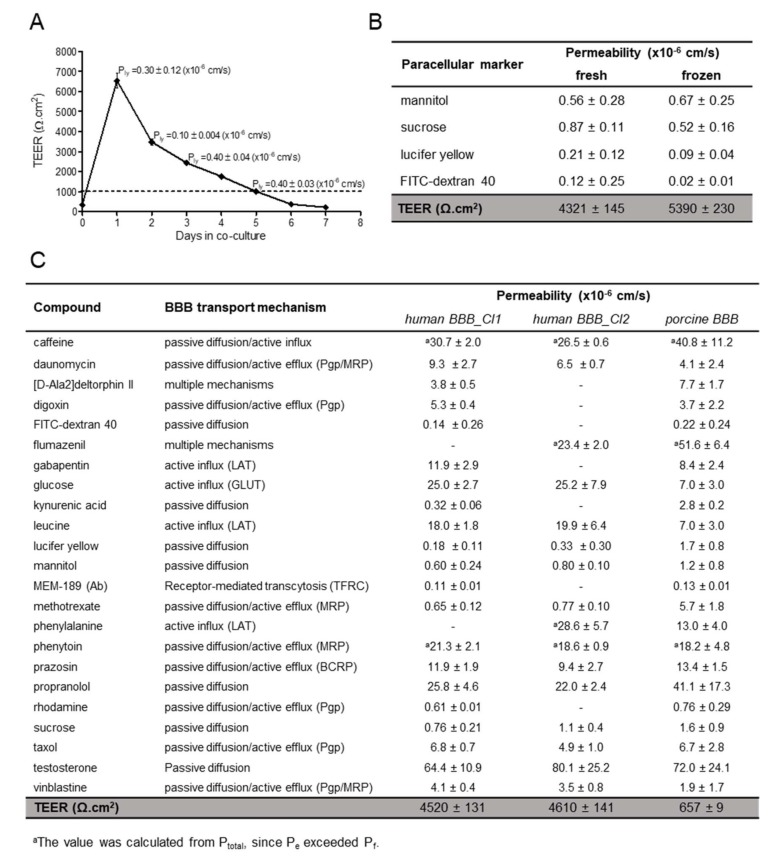
TEER and Permeability. (**A**) TEER measurements in hiPSC-derived BMECs (Cl1) were performed one day after seeding onto Transwells, just before co-culture with human astrocytes (d0) and every day thereafter, *n* = 3. Permeability values of the paracellular marker lucifer yellow were determined in triplicates and expressed as mean ± sd. (**B**) Paracellular markers permeability and TEER values at d1 co-culture in hiPSC-derived BMECs (Cl1) from fresh and frozen sources (cryopreserved at D8 of differentiation). Values of permeability are the mean ± sd from at least one determination with 3 biological replicates. TEER value is the mean ± SEM, with *N* = 96 (fresh) and *N* = 22 (frozen). (**C**) Summary of the permeability values in the in vitro human (Cl1 and Cl2) and porcine BBB models of compounds with different transport mechanisms. Values are the mean ± sd from at least one determination with 3 biological replicates. TEER value is the mean ± SEM, with *N* = 118 (Cl1); *N* = 100 (Cl2) and *N* = 701 (porcine). Porcine values, except D-Ala-deltorphin II, flumazenil and MEM-189, were previously reported [2].

After optimization of culture conditions and characterization of the expression of drug transporters, the ability of the cells to form a functional tight barrier was first investigated by measuring TEER. As reported in Figure 5A, hiPSC-derived BMECs reached values as high as 6500 Ω∙cm^2^ after one day of co-culture with human astrocytes. The high TEER decreased after two days of co-culture (d2) but it was maintained over 1000 Ω∙cm^2^ for a further 3–4 days, in the range thought to be physiologically relevant [32]. The paracellular transport of lucifer yellow was low (0.1–0.4 × 10^−6^ cm/s) and comparable to the in vivo value obtained in 15 µm pial post capillary venules in a rat model [33]. Above a threshold TEER value of 1000 Ω∙cm^2^ the hiPSC-derived BMECs displayed a comparable lucifer yellow permeability, indicating that this TEER value is sufficient to maintain the barrier stability (Figure 5A), as shown by the low permeability coefficient of the other small paracellular markers, such as sucrose and mannitol, and the larger marker FITC-dextran 40 (Figure 5B). The permeability of the paracellular marker lucifer yellow remained constant until d5. We usually performed transport studies at d1 and d2 with no significant differences in TEER values before and after incubation with the compounds (5%–20% of variation).

To expand the utility of the hiPSC-derived BMEC model to screening purposes, large quantities of consistently high-quality cells are required. To this end, we established a cryopreservation step for the endothelial progenitors before final maturation and differentiation in Transwells (at D8). We found that the BBB characteristics of hiPSC-derived BMECs were maintained after cryopreservation before the final selection on collagen/fibronectin matrix. The BBB properties of the hiPSC-derived BMEC models prepared from fresh or cryopreserved endothelial cells were comparable (Figure 5B), when considering their high TEER and very low paracellular permeability. Both fresh and cryopreserved hiPSC-derived BMECs were used, allowing the scale-up of the expansion of undifferentiated iPSCs followed by large-scale differentiation.

Similar to Cl1, the Cl2-derived BMECs exhibited a high TEER value and poor paracellular permeability by all paracellular markers tested, (<1.1 × 10^−6^ cm/s, Figure 5C), indicating also the robustness of the differentiation protocol. We previously measured the permeability of 54 commercial compounds using the porcine BBB model [2]; a selected subset of these compounds was tested in the human model, using two different hiPSC-derived BMECs: Cl1 and Cl2. As shown in Figure 5C, the human model allowed measurements of highly permeable compounds, such as testosterone and propranolol (passive diffusion), as already observed in the porcine model. The permeability coefficient of propranolol (22–26 × 10^−6^ cm/s) was in-line with the published values of 22.6 × 10^−6^ cm/s, and around 30 × 10^−6^ cm/s, reported by Delsing et al. [34] and Mantle et al. [35], respectively, measured in two different hiPSC-derived BMECs (r-iSPC 1j cells and IMR-90). Phenytoin, which is actively effluxed following passive entry, and caffeine, which has a dual mechanism of passive entry and active influx, were highly permeable in human BBB model as in the porcine model. This observation is consistent with previous results from Delsing et al. [34] who reported a phenytoin permeability value of 35.6 × 10^−6^ cm/s comparable to our measured values of 19-21 × 10^−6^ cm/s. Appelt-Menzel et al. [36] reported a caffeine permeability of 25.3 × 10^−6^ cm/s in IMR-90 cells, similar to the values we obtained of 27–31 × 10^−6^ cm/s.

**Figure 6:**
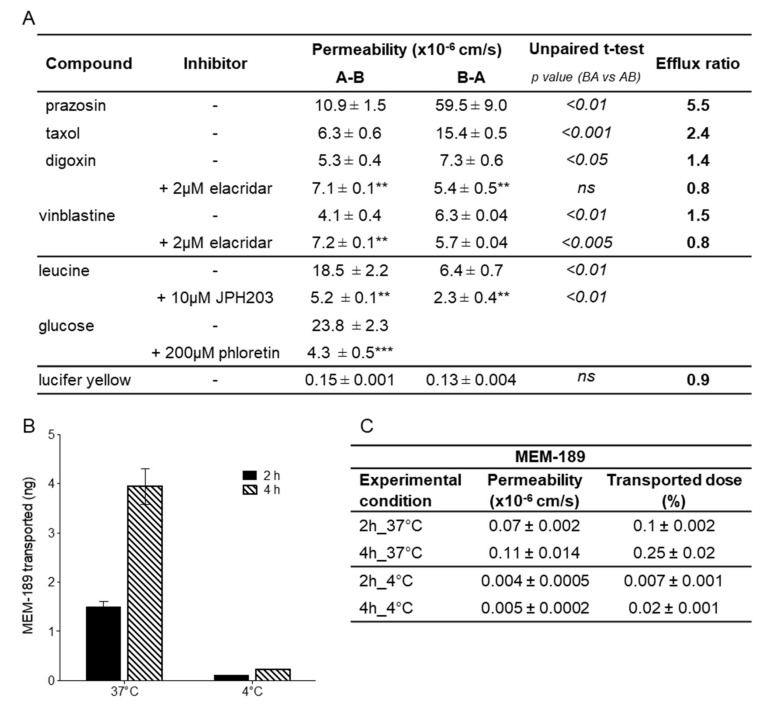
Polarised transport and transcytosis in hiPSC-derived BMECs. (**A**) Permeability of transported substrates across hiPSC-derived BMECs (Cl1) in presence of different inhibitors. Coefficients in the A-B and in the B-A directions are reported as mean ± sd of at least three biological replicates along with respective efflux ratio. Statistical differences between both sides is reported as the *p*-value; ns = not significant. The statistical analysis for the difference of permeability for the compound alone or in the presence of an inhibitor is indicated by asterisks: ***for *p* < 0.001, ** for *p* < 0.01 and * for *p* < 0.05. (**B**) hiPSC-derived BMECs support transcytosis of the anti-transferrin antibody MEM189. Amount of the antibody measured at 37 and 4 °C in the basal compartment after 2 and 4 h is the mean ± sd of duplicate determination of biological triplicates. (**C**) Permeability coefficient and percentage of transported dose of the anti-transferrin antibody MEM-189 in the experimental conditions as reported in B.

The known LAT substrate leucine was also highly permeable (18–20 × 10^−6^ cm/s), indicating the functional expression of this transporter, consistent with the measured levels of LAT1 (Figure 4). In the human model, the permeability of leucine was about three-fold higher than in the porcine model (see Figure 5C). In addition, the vectorial transport was three-fold higher in the AB direction than in the BA direction (Figure 6A) and reduced by about 70% in the presence of JPH203, a selective LAT1 inhibitor. This is consistent with the observed 75% inhibition in the uptake of the LAT1 specific substrate pregabalin, after LAT-1 mRNA silencing in the human endothelial cell line hCMEC/D3 [37]. Phenylalanine, another LAT-1 substrate, had a permeability value two-fold higher than in PBEC [2] similar to what was observed for leucine. A further LAT-1 substrate, gabapentin, had a moderate to high permeability with a coefficient value of 12 × 10^−6^ cm/s, ca. twice the permeability observed by Kurosawa at al. [20] and in the range of the porcine value.

Compounds known to be effluxed by PGP such as vinblastine and daunomycin, classified as having medium permeability, had permeability coefficients of 4 × 10^−6^ cm/s and 7–9 × 10^−6^ cm/s, respectively, and were more permeable in the human than in the porcine model (two-fold). Within the PGP substrates, taxol, digoxin, and rhodamine permeability values resembled the previously reported values in the porcine model (Figure 5C). The permeability value of rhodamine (0.6 × 10^−6^ cm/s) was also in agreement with the value reported (0.57 × 10^−6^ cm/s) in human BMECs derived from the BC1 iPS cell line [19]. Vinblastine was poorly transported probably because of the presence of efflux mechanisms. In fact, the functional polarity of the hiPSC-derived BMEC model was assessed by measuring the bi-directional transport of various known PGP substrates (Figure 6A). Unbalanced transport (A-B vs. B-A), along with increased apical to basolateral transport and decreased efflux ratio (ER) in the presence of elacridar, a PGP inhibitor, indicated that this efflux pump was functional and preferentially transported substrates in the brain to blood direction.

The BCRP substrate prazosin showed a coefficient of permeability of 9–12 × 10^−6^ cm/s, somewhat higher than the value of 4.3 × 10^−6^ cm/s reported by Lippmann [6], but similar to the value of 8.7 × 10^−6^ cm/s reported by Kurosawa’s group [20]; and could be clearly classified as an intermediate permeable compound like in the classification made using the porcine model. This compound is also susceptible to efflux as confirmed by the difference in permeability between the A-B direction versus the B-A direction (Figure 6A), with an ER of 5.5.

The opioid peptide D-Ala-deltorphin II showed moderate permeability in both models, although it was two-fold more permeable in PBECs. However, the moderate value of D-Ala-deltorphin II was in agreement with the BBB penetration in vivo in rodents, when given intravenously [38]. Kynurenic acid, a product of one branch of the kynurenine pathway of tryptophan metabolism, showed a low permeability in both systems, however, in the human model, its value was much closer to those of paracellular markers (0.3 in human vs. 2.8 in porcine). This can probably be attributed to the tighter barrier formed by the hiPSC-derived BMECs in comparison with the porcine model. Consistent with this, methotrexate was nearly eight times more permeable in the porcine model. The uptake of methotrexate is known to be a mix of passive diffusion and MRPs-mediated efflux. However, other MRP substrates had either unchanged (phenytoin) or even higher (daunomycin, vinblastine) permeability in the human model compare to the porcine model. This may indicate the presence of additional mechanisms responsible for their efflux. Chen et al. [39] have reported that MRP4 has a very broad substrate specificity and it can cause resistance to methotrexate exposure, while a study in mice reported the major role of BCRP over MRP2 in the methotrexate efflux from the brain [40]. Since BCRP was the most expressed ABC transporter in the human model, it might have a major role in limiting the permeability of methotrexate. Flumazenil, proposed to be a substrate of PGP [41], in our hands, was highly permeable as compared to other classical PGP substrates mentioned above, and to the BCRP substrate prasozin, but showed similar permeability values to the very recent one published by Le Roux et al. [22] and in-line with the porcine value. This would confirm the previous findings [42] that flumazenil is not a substrate for human PGP or BCRP.

Glucose displayed a more than 3.5-fold higher permeability (25 × 10^−6^ cm/s) in the human compared to the porcine model (7 × 10^−6^ cm/s, Figure 5C), and the transport was inhibited by the specific inhibitor phloretin by nearly 80% (Figure 6A). GLUT-1 (SLC2A1) was found at particularly high levels in endothelial cells and in the epithelial-like barriers of the brain [43] where it was expressed in both the luminal and abluminal membranes [44]. Using immunostaining, we observed higher expression in hiPSC-derived BMECs when compared to PBECs (Figure S2B).

Large molecules can cross the BBB via receptor-mediated transcytosis (RMT). Some BBB receptors, such as TFRC, low-density lipoprotein receptor-related proteins (LRP1, LRP2), and the insulin receptor (INSR), undergo RMT. TFRC has been shown to function as a molecular shuttle, transporting anti-TfR antibodies across the BBB, and for this reason has been extensively studied to facilitate enhanced transport of drugs to the brain. In order to assess the function of TFRC in our cellular system, we evaluated the transport of the high-affinity TfR antibody MEM-189 [45], which does not block the binding of transferrin in hiPSC-derived BMEC and PBECs. In the human BBB model (Figure 6B), the amount of MEM-189 found in the receiver compartment increased proportionally with time and more importantly, the amount at 4 °C represented less than 10% of the amount at 37 °C, indicating that the transport was mediated by an active mechanism. Permeability coefficients were very similar between human and porcine models (Figure 5C). In these transcytosis experiments, only a small fraction of the antibody (maximum of 0.25% after 4 h) was shuttled across the barrier, but this may be enough for potent molecules to achieve sufficient brain exposure to have a biological effect. The permeability of the paracellular marker sucrose (1.1 ± 0.6 × 10^−6^ cm/s), used as internal control, was in conformity with the mean value for the marker (Figure 5C), indicating the maintenance of the barrier properties and the absence of significant toxicity over the duration of the transport experiment. We have not investigated in details the intracellular trafficking of the antibody, but our data were in agreement with those reported by Sade et al. [45], where they demonstrated in the hCMEC/D3 model, transcytosis of MEM-189, but not for other anti-TfR antibodies (128.1, 13E4, M-A712). The permeability value of MEM-189, obtained in our model (0.11 × 10^−6^ cm/s), was also in line with the published value of 0.48 × 10^−6^ cm/s, obtained in the human BBB microfluidic model [46].

### 3.4. Drug Candidates Permeability Measured in hiPSC-BMECs

**Figure 7:**
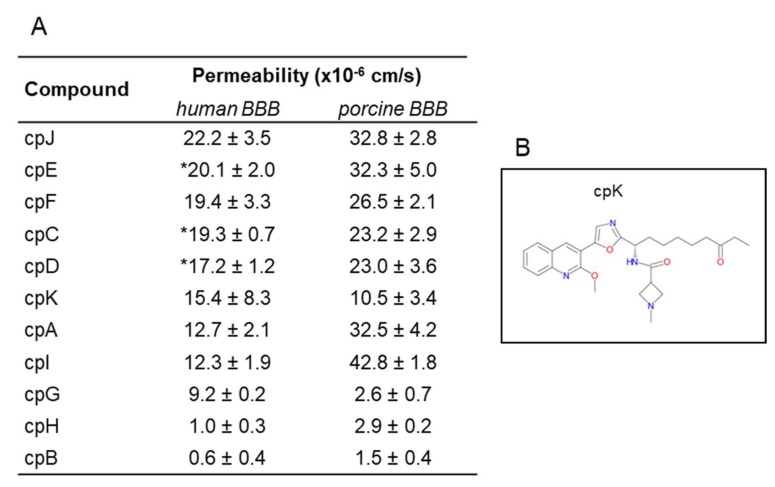
Permeability of investigational compounds. (**A**) Permeability values of investigational compounds in the in vitro human and porcine BBB models. Permeability values are expressed as mean ± sd from at least three biological replicates. * Compounds were tested in two clones (Cl1 and Cl2), values are expressed as mean ± SEM. (**B**) Chemical structure of cpK.

Once we established the hiPSC human BBB model and characterized its likeness to the actual human BBB, we aimed at verifying the utility of this model for screening experimental CNS drugs, testing investigational and proprietary molecules. These compounds were chosen on the basis of their permeability as previously measured in the PBEC model [2]. As reported in Figure 7A, the permeability values were very similar: for example, compound B (CpB) was poorly permeable in both human and porcine BBB models, and somewhat less permeable using the human model, reminiscent of the trend shown previously using the paracellular marker sucrose (Figure 5C). Likewise, CpH, a low permeable compound, resulted less permeable in human than in porcine model. Compounds C, D, E, F, and J showed high permeability in both models. While these molecules were less permeable in the human BBB, they would still be classified as medium to highly permeable compounds. The same is true for CpA and I, although the difference with the porcine values was higher: 2.5- and 3.5-fold, respectively. CpG is the only compound with significant higher permeability in human than in porcine model, reminiscent of the observed higher transport of glucose (GLUT-1) and leucine (LAT-1). The possibility that such a molecule could be substrate of a human transporter warrants further experiments.

We tested in our model a class I selective human HDAC inhibitor (CpK, Figure 7B), a preclinical development candidate that demonstrated proof-of-concept in an mdx mouse model for Duchenne muscular dystrophy and previously profiled in the porcine model [10]. This 2-methoxyquinoline derivative showed a medium permeability in both models, although the MPO score (a drug likeness central nervous system multiparameter optimization) is less than 3.8, slightly lower than the cut-off of 4 for probable permeable compound.

## 4. Discussion

Advances in iPSCs, genome engineering and differentiation protocols have rapidly expanded the use of iPSC-derived models as a preclinical tool for CNS compound profiling and drug development.

Effective treatment for the most common CNS diseases remains an unmet medical need and the BBB represents a significant impediment to their development. While the BBB regulates nutrient transport into the brain and helps it to function under physiological conditions, it also excludes the entry of most pharmaceuticals. Nearly all large-molecule therapeutics and greater than 95% of small-molecule drugs do not achieve adequate brain exposure [47]. The assessment of CNS-targeted drug delivery therefore requires a BBB model that displays limited paracellular permeability as well as expresses key polarized brain endothelial cell receptors and transporters.

Techniques to model the BBB in vitro are recognized as crucial to help to characterize the brain exposure of drug candidates prior to in vivo studies. In vitro systems are well suited to the study of biological processes in a more isolated context and have been most successfully used to elucidate mechanisms of action of specific transporters [48,49].

To date, multiple BBB models have been developed, ranging from cells in monolayers to more sophisticated spheroid and chip models. However the latest are generally not yet compatible with screening while the standard Transwell model offers the distinct advantage of higher throughput. This model enables access to both the apical and basolateral compartments for drug application and sampling, as well as the possibility to visualize the cells over the course of an experiment. To establish a more appropriate and highly relevant human assay system and to improve the translatability of in vitro data, we developed a human-based Transwell model which incorporates human astrocytes as a critical component of the neurovascular unit. The iPSCs were able to form a homogenous and functional population of brain endothelial cells that exhibited barrier, influx, and efflux BBB properties. We investigated the properties of two iPSC lines using the same differentiation protocol and they both were able to differentiate into brain-like endothelial cells with comparable characteristics, independently of the genetic background. Given the capacity of iPSC to generate nearly all cell types, a complete system with cells from the same genetic origin could be derived in a next step [36].

A very recent publication by Raphael Lis and collaborators [50] had raised doubts about the endothelial nature of in vitro BBB models derived from neuroendothelial differentiation of iPSC by opposition to the mesoendothelial differentiation, mainly on a transcriptome-based analysis. They reported that cells derived using neuroendothelial differentiation lack canonical endothelial markers such as VE-CADHERIN and PECAM1. At D8 we obtained a quite homogenous population for VWF expression that differed from the observed pattern, reported by Stebbins et al. [51] after staining with VE-CADHERIN and the neural marker NESTIN. This could be a result of the seeding density that can differ from cells lines. For example, Wilson et al. [52] reported for three different cells lines, the absence of the neural tracts when the cells were seeded at high density (>100,000 cells/cm^2^) followed by a low percentage of NESTIN-positive cells.

In our model, progenitor’s endothelial cells at D8 were directed towards to the endothelial phenotype based on the flow cytometry analysis of VWF. The resultant day ten (D10), BMEC-like cells were an homogeneous population expressing the endothelial markers such as VWF, CLAUDIN-5, PECAM1 and VE-CADHERIN, that conferred a BBB phenotype, in agreement with the tightness and the permeability restriction of the model described in this paper. The RNAseq data indicated the expression of additional CADHERINS such as E-CADHERIN and N-CADHERIN that has been reported to be expressed not only in brain microvessel endothelial cells, but in other cell types such as neural cells [53,54]. However, unlike N-CADHERIN, VE-CADHERIN is exclusive to endothelial cells because its role in the maintenance of cell-cell junction stabilization. The transcriptomic analysis indicated that the cells expressed, at high levels, additional markers belonging to an epithelial signature such as CLAUDIN-4 and CLAUDIN-7. However, differences between protein and RNA dynamics in terms of amplitude and temporal profiles have been a matter of debate and are still open questions. Data obtained by RNAseq do not necessarily show direct positive correlation with the protein levels due to the complex regulation of the gene expression at transcriptional, post-transcriptional and post-translational levels. [55]. As discussed in Section 3, a sort of discrepancy appears between mRNA and protein functional expression, as reported also by Delsing et al. [34]. In their differentiation protocol 1 (similar to the one described in this paper), CLAUDIN-5 was much less expressed at the RNA level but showed the right membrane localization at the cell borders by opposition to the protocol 2, having high RNA expression but more diffuse staining, indicative of a less mature phenotype. A deeper functional comparison of the two derivation protocols might also help to understand the relationship between mRNA, protein levels, and localization in highly and terminally differentiated cell types, such as brain-like endothelial cells.

To our knowledge this is the first study where an in vitro BBB model, established from iPSCs, has been characterized for the transport of as many as 23 molecules, with confirmation in a different clone indicating the reproducibility of the differentiation method. Li et al. [21] showed a good correlation between values obtained in a human iPSC-derived model with those obtained using an in situ brain perfusion method in rats for a panel of nine compounds, segregated into PGP/BCRP substrates and non substrates, proposing the assay as second-line screening for CNS drug candidates. We previously demonstrated a good correlation between permeability in the porcine model and in vivo brain partition [2], and here we show that permeability data derived from the human and porcine assays are very similar (the full test set of 23 compounds correlated with a coefficient of determination R^2^ of 0.8). Translatability from animal to human remains an important issue, since recent studies have highlighted significant species differences in the BBB, with the most important being related to efflux pumps and transporters [56]. Overall, porcine and human iPSC-derived BBB models shared similar characteristics. A major difference is the relevance of the efflux pump, such as BCRP in transporting out compounds and the higher transport activity of some SLCs in the human model. We found higher transport rates of amino acids by LAT1 (SLC7A5) and glucose by GLUT1 (SLC2A1) transporters in the human model. However, that could be due to a difference in the level of expression in the two species. To avoid misleading results and provide a best-fit model, in vitro data of known CNS compounds should be correlated with human pharmacokinetic data obtained using imaging tools, such as PET. Very recently, Le Roux et al. [22] clinically validated the iPSC-derived BBB model by comparing in vitro permeability with brain permeability derived from PET (positron emission tomography) data of 10 compounds.

The model described here has key features of the BBB, including strong monolayer tightness and restrictive paracellular permeability, functional expression of BBB-specific transport processes, and polarized transport. In addition, the model can be used to assess passage of large biopharmaceuticals such as antibodies, and it is suitable for reproducible mechanistic studies in a drug discovery setting. The RNAseq analysis verified the expression of all the important BBB related genes in the human iPSC derived endothelial cells comprising efflux and influx transporters.

The SLC superfamily of transporters are critical drug uptake transporters at the BBB. The SLC superfamily lists about 52 distinctive families with nearly 400 unique transporter genes in the human genome of which more than 60% were detected in the hiPSC-derived BMEC model. This highlights the utility of using this platform as a dual-tracking screen in CNS development programs, as proposed originally by Pardridge [57]. Although in silico models can be used for the qualitative BBB permeability estimation using various physicochemical descriptors, these may not be useful to predict the active BBB transport through efflux pumps, carriers, and receptors.

In addition to investigation of transport function, the human BBB model will allow studies to examine disease-related differences in BBB function based on stem cells derived from healthy individuals and those affected by CNS diseases. The development of models from patient-derived iPSCs provide the opportunity to study disease biology and design more effective screening paradigms. Katt et al. [58] explored the impact of mutations associated with neurodegenerative diseases in the functionality of the BBB. They showed that at least one BBB property, among TEER, paracellular permeability, transcellular permeability (glucose), and rhodamine efflux ratio, was affected. In particular, endothelial cells derived from HD patients showed increased transport of rhodamine, suggesting possible PGP dysfunction. These data are in agreement with the correlation between CAG repeat length and increased permeability of rhodamine reported by the group of Thompson [7]. To potentiate the discovery of drugs tailored to patient needs, model selection parameters are critical for the evaluation of drug properties and, specifically for the brain, of transport behavior, because the disease in question may affect the barrier properties of the BBB. In view of the above, iPSC patient-derived BBB models provides a unique opportunity to identify disease signatures and can compensate for the lack of predictive in vitro human systems for drug discovery and delivery.

## Supplementary Materials

The following are available online at https://www.mdpi.com/2073-4409/9/4/994/s1, Figure S1. characterization of hiPSCs; Figure S2. (A) Final differentiation of hiPSCs into brain endothelial cells (Cl2), (B) Representative immunofluorescence staining of GLUT1 at day 2 of co-culture of PBECs with rat astrocytes. Table S1. Antibodies used in this study; Table S2. Genes evaluated in this study by TaqMan assays.
