# Supplementary material for: Establishment of an in Vitro Human Blood-Brain Barrier Model Derived from Induced Pluripotent Stem Cells and Comparison to a Porcine Cell-Based System
Source: Cells. 2020 Apr 16;9(4):994. doi: 10.3390/cells9040994 (PMC7226989; doi:10.3390/cells9040994)
Supplement: Supplementary file 1 [file cells-09-00994-s001.pdf]

Supplemental Information for

Establishment of an *in vitro* human blood-brain barrier model derived from induced pluripotent stem cells and comparison to a porcine cell-based system

by

Annalise Di Marco*, Domenico Vignone, Odalys Gonzalez Paz, Ivan Fini, Maria Rosaria Battista, Antonella Cellucci, Elena Bracacel, Giulio Auciello, Maria Veneziano, Vinod Khetarpal, Mark Rose, Alessandro Rosa, Isabelle Gloaguen, Edith Monteagudo, Todd Herbst, Celia Dominguez, and Ignacio Muñoz-Sanjuán*

* To whom correspondence should be addressed: a.dimarco@irbm.com; ignacio.munoz@chdifoundation.org

This PDF file includes:

(1) Supplemental Figures S1 and S2 and legends (page 2)

(2) Supplemental Tables S1 and S2 (pages 3 to 4)

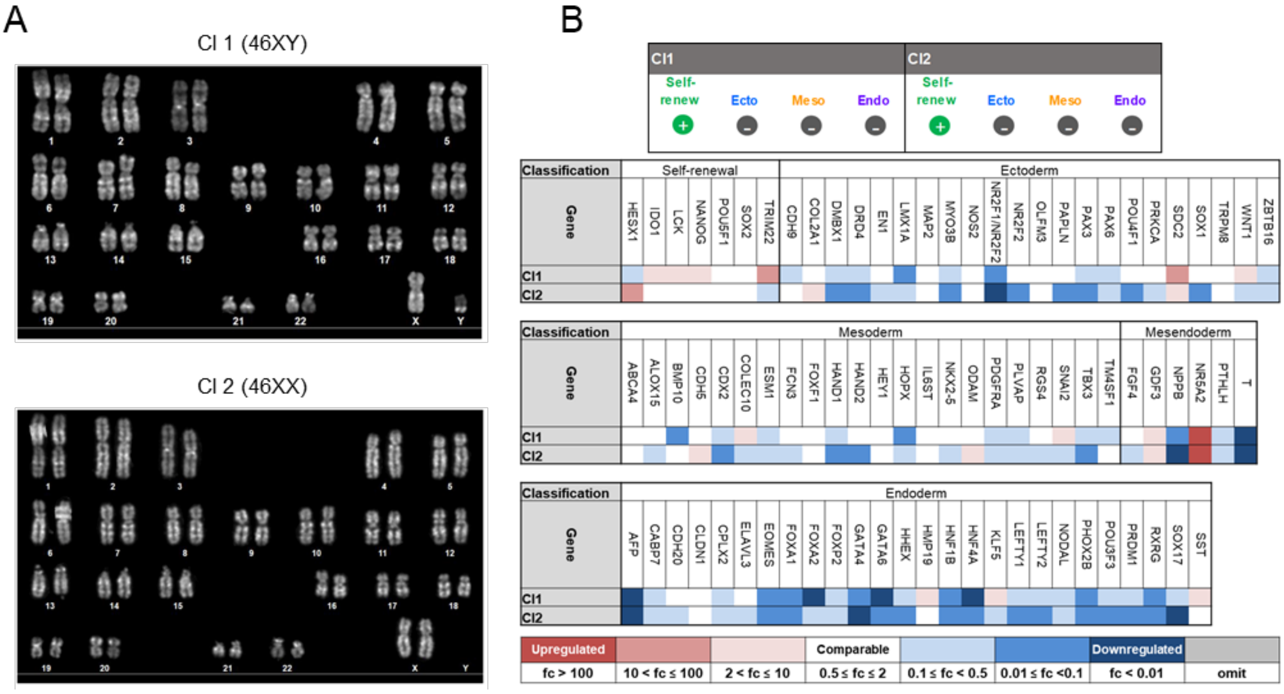

Figure S1. Characterization of human iPSCs. Related to Figure 1. (A) Representative image of the Cl1 at passage 27 and Cl2 at passage 30 showing a normal karyotype with no chromosomal aberration. (B) The pluripotency of the clones as well as their self-renewal ability were confirmed by pluripotency score card analysis

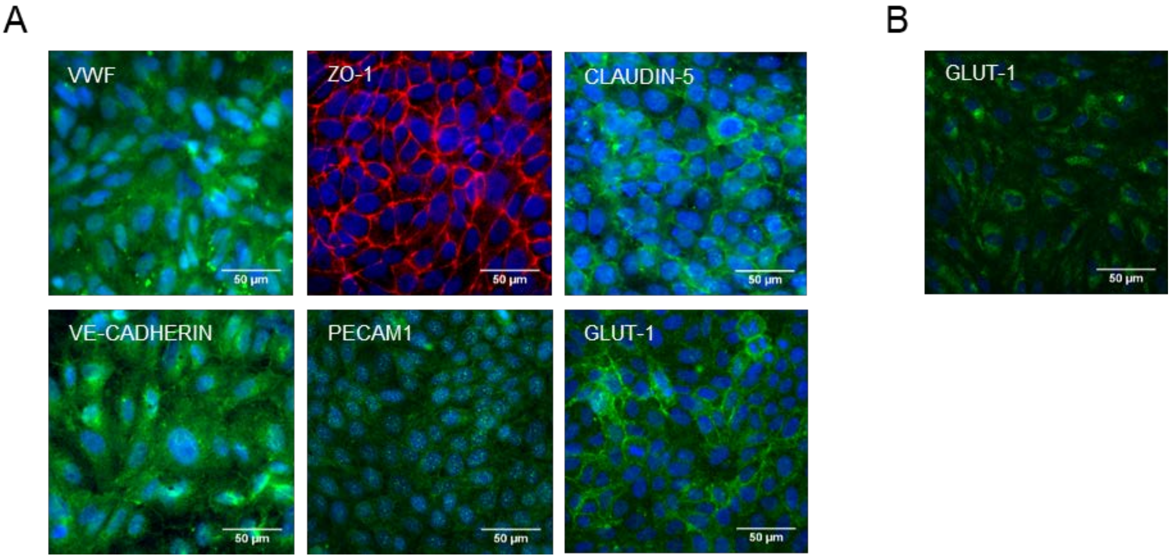

Figure S2. (A) Final differentiation of hiPSCs into induced brain endothelial cells (Cl2). Related to Figure 3A. Representative immunofluorescence staining, at d1 co-culture in Transwell filters, demonstrating the expression of endothelial relevant proteins: VWF, ZO-1, CLAUDIN-5, VE-CADHERIN, PECAM1, and GLUT-1. (B) Representative immunofluorescence staining of GLUT1 at day 2 co-culture of PBECs with rat astrocytes.

The nuclei were stained with Hoechst (blue). Scale bar represented 50 µm.

Table S1. Antibodies used in this study. Related to flow cytometry, immunocytochemistry and western blot.

Flow Cytometry

**Target epitope**	**Species**	**Type**	**Label**	**RRID***	**Vendor**	**Cat nb**	**Dilution**
OCT4	mouse	monoclonal	Allophycocyanin	AB_10718265	R&D Systems	IC6344A	10µl/10^6cells
Match IgG_{2A}	mouse	monoclonal	Allophycocyanin	AB_10673794	R&D Systems	IC003A	10µl/10^6cells

Immunofluorescence

**Target epitope**	**Species**	**Type**	**Label**	**RRID***	**Vendor**	**Cat nb**	**Dilution**
OCT4	rabbit	monoclonal		AB_10979606	Thermo Fisher	MA5-14845	1:400
NANOG	mousse	monoclonal		AB_2536677	Thermo Fisher	MA1-017	1:100
SOX2	mouse	monoclonal		AB_2536667	Thermo Fisher	MA1-014	1:200
VWF	rabbit	polyclonal		AB_2315602	Agilent (Dako)	A008202-5	1:500
CLAUDIN-5	rabbit	polyclonal		AB_2533157	Thermo Fisher	34-1600	1:25
OCCLUDIN	rabbit	polyclonal		AB_2533977	Thermo Fisher	71-500	1:25
ZO-1	mouse	monoclonal		AB_2533147	Thermo Fisher	33-9100	1:200
PECAM1 (CD31)	rabbit	polyclonal		AB_720501	Lab Vision	RB-10333	1:50
VE-Cadherin	goat	polyclonal		AB_355726	R&D Systems	AF938	10 µg/ml
GLUT-1	mouse	monoclonal		AB_10979643	Thermo Fisher	MA5-11315	1:50
GLUT-3	rabbit	polyclonal		AB_732609	Abcam	Ab41525	1:100
PGP (Mdr1)	mouse	monoclonal		AB_11026685	NevusBio	NBP1-90291	1:50
BCRP (ABCG2)	mouse	monoclonal		AB_95060	Sigma	MAB4155	1:50
Anti-rabbit IgG (H+L)	goat	polyclonal	Alexa Fluor 488	AB_2576217	Thermo Fisher	A-11034	1:3000
Anti-rabbit IgG (H+L) cross-adsorbed	goat	polyclonal	Alexa Fluor 568	AB_143157	Thermo Fisher	A-11011	1:3000
Anti-mouse IgG (H+L) cross-adsorbed	rabbit	polyclonal	Alexa Fluor 594	AB_2534109	Thermo Fisher	A-11062	1:3000
Anti-goat	rabbit	polyclonal	Alexa Fluor 488	AB_2534122	Thermo Fisher	A-11078	1:3000

Western Blot

**Target epitope**	**Species**	**Type**	**Label**	**RRID***	**Vendor**	**Cat nb**	**Dilution**
CLAUDIN-5	rabbit	polyclonal		AB_2533157	Thermo Fisher	34-1600	1:500
PGP	mouse	monoclonal		AB_2625786	OriGene	TA801055	1:500
PECAM1	rabbit	polyclonal		AB_720501	Lab Vision	RB-10333	1:500
BCRP	mouse	monoclonal		AB_95060	Sigma	MAB4155	1:500
Actin	rabbit	polyclonal		AB_476693	Sigma	A2066	1:2000
Actin	mouse	monoclonal		AB_476697	Sigma	A2228	1:2000
Anti-rabbit IgG (H+L) cross-absorbed	goat	polyclonal	Alexa Fluor 800	AB_2633284	Thermo Fisher	A32735	1:5000
Anti-mouse IgG (H+L) cross-absorbed	goat	polyclonal	Alexa Fluor 680	AB_2633278	Thermo Fisher	A32729	1:5000

*: Research resource identifier

Table S2. Genes evaluated in this study and the TaqMan assays used for mRNA quantification. Related to quantification by real-time qRT-PCR.

**Symbol**	**Alias**	**Gene name**	**ID** (NCBI)	**Assay ID** (ThermoFisher)
GAPDH	G3PD; GAPD; HEL-S-162eP	glyceraldehyde-3-phosphate dehydrogenase	2597	Hs02758991_g1
NANOG	-	nanog homeobox	79923	Hs02387400_g1
POU5F1	**OCT4**, OCT3, OTF3, OTF4	POU class 5 homeobox 1	5460	Hs04260367_g1
SNAI2	SLUG, SLUGH, SLUGH1, SNAIL2, WS2D	snail family transcriptional repressor 2	6591	Hs00161904_m1
